# GLUT1 gene is a potential hypoxic marker in colorectal cancer patients

**DOI:** 10.1186/1471-2407-9-241

**Published:** 2009-07-20

**Authors:** Fu-Yen Chung, Ming-Yii Huang, Ching-Sheng Yeh, Hui-Jen Chang, Tian-Lu Cheng, Li-Chen Yen, Jaw-Yuan Wang, Shiu-Ru Lin

**Affiliations:** 1 Graduate Institute of Medicine, Kaohsiung Medical University, Kaohsiung, Taiwan, Republic of China; 2 Department of Radiation Oncology, Kaohsiung Medical University Hospital, Kaohsiung, Taiwan, Republic of China; 3 Department of Radiation Oncology, Faculty of Medicine, College of Medicine, Kaohsiung Medical University, Kaohsiung, Taiwan, Republic of China; 4 Faculty of Biomedical Science and Environmental Biology, Kaohsiung Medical University, Kaohsiung, Taiwan, Republic of China; 5 Division of Gastrointestinal and General Surgery, Department of Surgery, Kaohsiung Medical University Hospital, Kaohsiung, Taiwan, Republic of China; 6 Department of Surgery, Faculty of Medicine, College of Medicine, Kaohsiung Medical University, Kaohsiung, Taiwan, Republic of China; 7 Bio-medical Technology Developmental Center, Fooyin University, Kaohsiung Hsien, Taiwan, Republic of China; 8 Department of Medical Technology, School of Medical and Health Sciences, Fooyin University, Kaohsiung Hsien, Taiwan, Republic of China; 9 Department of Medical Research, Fooyin University Hospital, Ping-Tung Hsien, Taiwan, Republic of China

## Abstract

**Background:**

Tumor hypoxia is an important factor related to tumor resistance to radiotherapy and chemotherapy. This study investigated molecules synthesized in colorectal cancer cells during hypoxia to explore the possibility of developing molecular probes capable of detecting cell death and/or the efficiency of radiotherapy and chemotherapy.

**Methods:**

At first, we incubated two human colorectal adenocarcinoma cell lines SW480 (UICC stage II) and SW620 (UICC stage III) cells in hypoxic (≤2% O_2_, 93% N_2_, and 5% CO_2_) and normoxic conditions (20% O_2_, 75% N_2_, and 5% CO_2_) for 24 h and 48 h. The relative expression ratio of GLUT1 mRNA in hypoxic conditions was analyzed by RT-PCR. Ten cancerous tissues collected from human colorectal cancer patients were examined. HIF-1α and HIF-2α levels were measured to indicate the degree of hypoxia, and gene expression under hypoxic conditions was determined. As a comparison, HIF-1α, HIF-2α, and GLUT1 levels were measured in the peripheral blood of 100 CRC patients.

**Results:**

Hypoxia-induced lactate was found to be elevated 3.24- to 3.36-fold in SW480 cells, and 3.06- to 3.17-fold in SW620 cells. The increased relative expression ratio of GLUT1 mRNA, under hypoxic conditions was higher in SW620 cells (1.39- to 1.72-fold elevation) than in SW480 cells (1.24- to 1.66-fold elevation). HIF-1α and HIF-2α levels were elevated and GLUT1 genes were significantly overexpressed in CRC tissue specimens. The elevated ratio of GLUT1 was higher in stage III and IV CRC tissue specimens than in the stage I and II (2.97–4.73 versus 1.44–2.11). GLUT1 mRNA was also increased in the peripheral blood of stage II and III CRC patients as compared to stage I patients, suggesting that GLUT1 may serve as a hypoxic indicator in CRC patients.

**Conclusion:**

In conclusion, this study demonstrated that GLUT1 has the potential to be employed as a molecular marker to indicate the degree of hypoxia experienced by tumors circulating in the blood of cancer patients.

## Background

Hypoxia is an important factor in tumor biology as it plays a critical role in resistance to radiation therapy and chemotherapy [1,2]. Hypoxia is tightly correlated with aggressive tumor behavior, including angiogenesis, aggressiveness, local recurrence, and distant metastasis [3,4]. Hypoxia appears to be a poor prognostic factor for several cancers, including colorectal, cervix, head and neck, and prostate [5-8]. Despite the importance of tumor oxygenation status in both therapy and prediction of disease progression, to date, there has been no consensus as to the best method for the detection of hypoxia [4]. Currently available techniques are classified broadly into direct invasive methods, direct non-invasive methods, and measurement of surrogate endogenous or chemical markers of tumor oxygenation. Of these methods, the Eppendorf pO_2 _histograph is considered the "gold standard" for determination of intratumoral oxygen tensions, but this method is invasive [9-11]. Recent studies have focused on molecular markers of hypoxia. The chemical hypoxia marker, pimonidazole, and expression of the endogenous hypoxia markers (carbonic anhydrase IX, hypoxia-inducible factor 1α, osteopontin, ephrin A1, galectin-1, and lysyl oxidase) are used to detect hypoxia by immunohistochemical staining of tumor biopsy tissues [5,6,12]. Lactate is the end product of glycolysis; and in 1996, Terpstra reported that *in vivo *measurements of tumor lactate concentration may provide valuable information about tumor metabolism [13]. Our previous study showed that the glycolytic pathway and glycolysis-related genes may play an important role in the tumorigenesis of CRC. Cell line studies identified 8 genes, including GLUT1, HK1, GPI, GAPD, PGK1, PGK2, ENO2, and PKM2, that have a high level of expression. Furthermore, Hypoxia-inducible factor 1 (HIF-1) has been found to target the transcription of over 60 genes involved in many aspects of cancer biology, including cell survival, glucose metabolism, cell invasion and angiogenesis [14]. These genes might provide a reference to the future clinical diagnosis of CRC and the pharmaceutical efficacy of treatment.

In publicly acknowledged clinical experiences with CRC patients, regular clinical biochemical tests and imaging studies have been employed as tools for post-surgical follow-up. Of these, carcinoembryonic antigen (CEA) is the most commonly used tumor marker for the detection of non-symptomatic recurrence. However, recent studies have pointed out that the sensitivity of CEA and CA 19-9 are only 30% and 18%, respectively [15]. Although CEA detection in the serum and tumor tissue of colorectal cancer (CRC) patients is the most commonly used marker for the diagnosis and evaluation of prognosis or recurrence after treatment, its role remains controversial [16]. In terms of tools for imaging studies, F-18-fluorodeoxyglucose-positron emission tomography (FDG-PET) can be utilized for early assessment of the chemotherapy response in patients with advanced colorectal cancer [17]. However, FDG-PET is a functional testing tool, so other structural imaging tools, such as computed tomography (CT) and magnetic resonance imaging (MRI), need to be employed in conjunction with FDG-PET for more precise location of the tumor. Moreover, inflammation, activity of the intestinal tract itself or constipation can often cause false-positives in FDG-PET studies. Using CT in the preoperative staging of colorectal cancer, even if controversial, may be useful for planning surgery and/or neoadjuvant therapy [18]. CT is one of the routinely used methods for detecting local recurrence, but recurrence in the pelvis and post-surgical fibrosis are hard to differentiate with this technique. Therefore, there are shortfalls in current methodologies being offered. Both imaging tools and chemotherapy have their own inherent inconveniences and deficiencies, especially in providing evidence for the therapeutic efficacy of target drugs.

According to the report by Weidner *et al*. [19], active angiogenesis may occur in prostate cancer tissue growing to 2 mm in diameter. In a metastatic state, each gram of tumor may shed approximately 10^6 ^cells into the blood vessel [20]. Since blood sampling is relatively easy, we wish to discover a molecular marker that could be used to assess the hypoxia level of peripheral circulating cancer cells in the blood specimens of cancer patients. To date, however, no correlation between the expression levels of these mRNA markers and the cancer progression stages of CRC patients has been determined. In the present study, we investigate the correlation of hypoxia-related genes and glycolysis-related genes and clinical prognosis in CRC patients in Taiwan. We present the correlation between lactate concentrations with hypoxia status in CRC cell lines under hypoxic conditions; and in parallel, we demonstrate the expression of the transcription factor HIF-1α and of the GLUT1 (a glucose transporter protein) mRNA by RT-PCR. We exploited membrane array to quantitatively analyze the expression of GLUT1 mRNA markers in the circulating tumor cells of patients with stage-II and -III CRC. The aim of this innovative diagnosis technique established in this study is to achieve early CRC detection and improve the efficacy of therapies in managing this malignancy.

## Methods

### Colorectal cancer cell lines

Two human colorectal adenocarcinoma cell lines, SW480 (UICC stage II) and SW620 (UICC stage III), were obtained from American-Type Culture Collection (CCL-228 and CCL-227; ATCC, Rockville, MD, USA) and used for this study. Cells were maintained in Leibovitz's L-15 medium (Gibco Life Sciences, BRL, Grand Island, NY, USA) and supplemented with 10% (v/v) fetal bovine serum (FBS) at 37°C in humidified atmospheric air without CO_2 _addition. Cell density was maintained in approximately 100,000 cells/mL medium in T-25 flasks (Corning, NY, USA). When the cells had grown to a confluent monolayer, they were harvested by washing the dishes once with phosphate-buffered saline (PBS), pH 7.3, and then incubated in PBS containing 0.53 mmol/L EDTA and 0.05% trypsin (Gibco) for 10–15 min at 37°C. The trypsinized cells were counted, and cell viability was assessed by trypan blue dye exclusion. Then, the cells were incubated for 24 and 48 h under two different oxygenated conditions (hypoxia: ≤2% O_2_, 93% N_2_, 5% CO_2_; normoxia: 20% O_2_, 75% N_2_, 5% CO_2_).

### Lactate concentration measurements

Cells were rinsed twice with phosphate-buffered saline, fresh medium was added to the cultures with and without each of the glucose analogs tested (2-Deoxy-D-glucose (2DG), at the indicated concentrations, and the cells were incubated at 37°C in humidified atmospheric air without CO_2 _addition for 24 and 48 h. Then, the media were collected for lactate concentration determination. The supernatant was centrifuged three times to yield a final clear supernatant. Aliquots of the culture medium were removed for content analysis. The absorbance was measured at 540 nm with a Multiskan^® ^EX ELISA Reader (Thermo Electron Corp., Finland) using a linear range of standard lactate concentrations according to the procedures recommended by the manufacturer (Trinity Biotech, Jamestown, NY). The intra-assay coefficient of variation was 1.1%. Samples were analyzed in triplicate. The relative concentration of lactate was examined after normoxia and hypoxia incubations for either 24 h or 48 h, respectively.

### Reverse transcriptase-polymerase chain reaction (RT-PCR)

RNA was extracted from colorectal cancer cell lines using ISOGEN™ (Nippon Gene Co., Ltd., Toyama, Japan) and QIAmp^® ^RNA Blood Mini Kit (Qiagen Inc., Valencia, CA). Total RNA (20 μg) was reverse transcribed to cDNA and two microliters of each of these cDNA samples were used for each PCR reaction. Two target genes, HIF-1α and GLUT1, were detected by RT-PCR. Sequences of the oligonucleotide primers were designed according a PCR primer selection program based on primer 3 at http://frodo.wi.mit.edu. Also, β-actin primers were added as internal controls to correct for the differences in total RNA amounts between the SW480 and SW620 cells. Each RT-PCR reaction mixture contained 1 × PCR buffer (10 mmol/L Tris-HCL, pH 8.3, 50 mmol/L KCL, 2 mmol/L MgCl_2_), 50 μmol/L dNTP, 0.1 μmol/L sense and antisense primers for target genes, and 0.01 μmol/L sense and antisense primers for β-actin, and 2.5 U Taq DNA polymerase in a total volume of 50 μL. PCR amplification was carried out in a GeneAmp 2700 thermocycler (Applied Biosystems, Foster City, CA), under the following conditions: initial denaturation at 95°C for 5 min; 33–35 cycles at 94°C for 30 s, at 60°C for 30 s, at 72°C for 1 min, and then at 72°C for 5 min for the final extension. PCR products were analyzed in 3% agarose gel containing 0.5 μg/mL ethidium bromide. The signals on UV transilluminator for each target gene and β-actin expression levels were scanned with a computing laser densitometer (Alpha Inotech, San Leandro, CA, USA) to calculate the relative mRNA density. Additional File 1 lists the primer sequences, PCR conditions, and sizes of PCR products.

### Tissue and blood samples

Ten CRC patients, ages ranging from 32 to 68 years (mean age: 51.5 ± 13.1 years), were selected randomly from a patient group at the Department of Surgery of Kaohsiung Medical University Hospital between May 2006 and March 2008. None of these patients had received any preoperative radiotherapy or chemotherapy. According to the prospective protocol, the tumors and all of the lymph nodes were cut at different levels and embedded in paraffin, and sections were taken for routine H&E staining. Senior pathologists checked all of these slides and documented the pathological characteristics of the tumor and lymph nodes. The tumor stage was defined according to the criteria of the American Joint Commission on Cancer/International Union Against Cancer (AJCC/UICC, 2002). Patients' characteristics are depicted in Additional File 2. Each tissue sample was snap-frozen in liquid nitrogen immediately after surgery, and stored at -80°C. Between July 2006 and May 2008, peripheral blood samples of 100 pre-operative CRC patients (mean age: 57.4 ± 13.4 years) were collected for genotype analysis. Five millilitres of peripheral blood were collected with test tubes containing anticoagulant sodium citrate from each of the enrolled patients before receiving any anticancer treatment, e.g., surgery, radiotherapy or chemotherapy. To avoid the contamination of skin cells, the sampled blood was taken via an intravenous catheter, and the first few millilitres of blood were discarded. Total RNA was immediately extracted from the peripheral whole blood, and then served as templates for cDNA synthesis. Written informed consent was obtained from all subjects and/or guardians for the use of their tissue and blood samples. Tissue and blood acquisition and subsequent use were approved by the institutional review board of Kaohsiung Medical University.

### Total RNA extraction and first strand cDNA synthesis

Total RNA was extracted from colorectal cancer patient's tissue and peripheral blood specimens with ISOGEN™ (Nippon Gene, Toyama, Japan) and QIAmp^® ^RNA Blood Mini Kit (Qiagen Inc., Valencia, CA) according to the manufacturer's instructions [21]. The RNA concentration was determined spectrophotometrically on the basis of absorbance at 260 nm (Beckman, DU800, USA). First-strand cDNA was synthesized from total RNA using a RT-PCR kit (Promega Corp., Madison, WI). The reverse transcription was carried out in a reaction mixture consisting of 1× Transcription Optimized 5× Buffer, 25 μg/ml oligo (dT)-15mer primer, 100 mmol/L PCR Nucleotide Mix, 200 μmol/L M-MLV Reverse Transcriptase, and 25 μL of Recombinant RNasin^® ^Ribonuclease Inhibitor (Promega). The reaction mixtures were incubated at 42°C for longer than 2 h, heated to 95°C for 5 min, and then stored at -80°C until analysis. Total RNA (20 μg) was reverse transcribed to cDNA.

### Membrane array preparation for hypoxia- and glycolysis-associated gene expression

**Figure 1:**
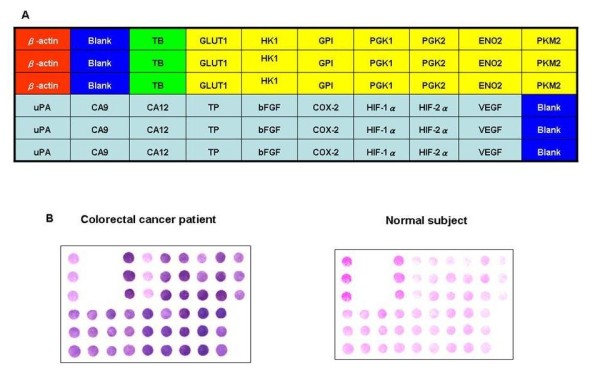
**Schematic representation of membrane array and comparison of gene expression patterns between a human colorectal cancer patient (Case No. 6) and healthy control**. (A) Schematic representation of membrane array with 16 target genes, one housekeeping gene (β-actin), one bacteria gene (TB), and blank control. Sixteen target genes (i.e., GLUT1, HK1, GPI, PGK1, PGK2, ENO2, PKM2, uPA, CA9, CA12, TP, bFGF, COX-2, HIF-1α, HIF-2α, and VEGF). The position of the correlation of the blank and positive control (β-actin) as well as the negative control (*Mycobacterium tuberculosis*; TB) in the nylon membrane are pointed out by spots. The relevant positions are the red area (β-actin), blue area (blank), green area (TB), yellow area (glycolysis-related genes), and pink area (hypoxia-related genes). (B) A triplicate set of 16 molecular markers for colorectal cancer was blotted on the nylon membrane. In addition, a housekeeping gene and a bacterial gene serving as positive and negative controls, respectively, were also blotted on the membrane.

The procedure of the membrane-array method for the detection of CRC-related genes was performed based on our previous work [22-24]. Visual OMP3 (Oligonucleotide Modeling Platform, DNA Software, Ann Arbor, MN) was used to design probes for each target gene and β-actin, the latter of which was used as an internal control (Additional File 3). The probe selection criteria included strong mismatch discrimination, minimal or no secondary structure, signal strength at the assay temperature, and lack of cross-hybridization. The oligonucleotide probes were then synthesized according to the designed sequences, purified, and controlled before being grafted onto the substracts. The newly synthesized oligonucleotide fragments were dissolved in distilled water to a concentration of 20 mM, applied to a BioJet Plus 3000 nL dispensing system (BioDot, Irvine, CA), which blotted the sixteen target oligonucleotides and TB (Mycobacterium tuberculosis) and the β-actin control sequentially (0.05 μL per spot and 1.5 mm between spots) on SuPerCharge nylon membrane (Schleicher and Schuell, Dassel, Germany) in triplicate. Dimethyl sulfoxide (DMSO) was also dispensed onto the membrane as a blank control (Figure 1A). After rapid drying and cross-linking procedures, the preparation of membrane array for hypoxia- and glycolysis-associated gene expression was accomplished [25]. A triplicate set of 16 molecular markers of colorectal cancer was blotted on nylon membrane. In addition, the housekeeping gene was β-actin while the bacterial gene was derived from Mycobacterium tuberculosis. Both served as positive and negative controls, respectively, and blotted on the membrane. The membrane array was used to analyze the gene expression of tumor and normal counterpart tissue in 10 CRC patients and 100 peripheral blood specimens.

### Preparation of digoxigenin-labeled cDNA targets and hybridization

First-strand cDNA targets for hybridization were produced by using SuperScript reverse transcriptase (Gibco-BRL, Gaithersburg, MD) in the presence of digoxigenin (DIG)-labeled dUTP (Roche Diagnostics GmbH, Penzberg, Germany). After the pre-hybridization procedures, the gene chips were subjected to hybridization. The lifts were covered with Express Hyb Hybridization Solution (BD Biosciences, Palo Alto, CA) containing DIG-11-UTP-labeled cDNA probes. After the washing and blocking steps, the arrays were incubated with alkaline phosphatase-conjugated anti-digoxigenin antibody (Roche Diagnostics). For hybridization, the arrays were incubated at 42°C for 12 h in a humidity chamber. For signal detection, the gene chips were incubated in chromogen solution containing nitroblue-tetrazolium and 5-bromo-4-chloro-3-indoyl-phosphate (NBT/BCIP) for 15 min. The hybridized arrays were then scanned with an Epson Perfection 1670 flatbed scanner (SEIKO EPSON Corp., Nagano-ken, Japan). Subsequent quantification analysis of each spot's intensity was carried out using AlphaEase^® ^FC software (Alpha Innotech Corp., San Leandro, CA). Spots consistently carrying a factor of two or more were considered as differentially expressed. These array analysis tools facilitated the measurement of relative gray levels of objects in a uniformly spaced array, such as dot blots. A deformable template extracted the gene spots and quantified their expression levels by determining the integrated intensity of each spot after background subtraction. The fold ratio for each gene was calculated as follows: spot intensity ratio = mean intensity of target gene/mean intensity of β-actin. Figure 1A provides the schematic representation of the membrane array with 16 target genes, one housekeeping gene (β-actin), one bacterial gene (TB), and the blank control.

### Statistical analysis

All data were analyzed using the Statistical Package for Social Sciences Version 11.5 software (SPSS Inc., Chicago, IL). Data are presented as means ± SE, and *P *values were determined by unpaired Student's t test. Furthermore, the two-sided Pearson χ^2 ^test was used to analyze the differences in the over-expression of the genotypes between different age groups, gender and stages. A *P *value of less than 0.05 was considered statistically significant.

## Results

### Correlation between Lactate concentrations with hypoxia status

**Figure 2:**
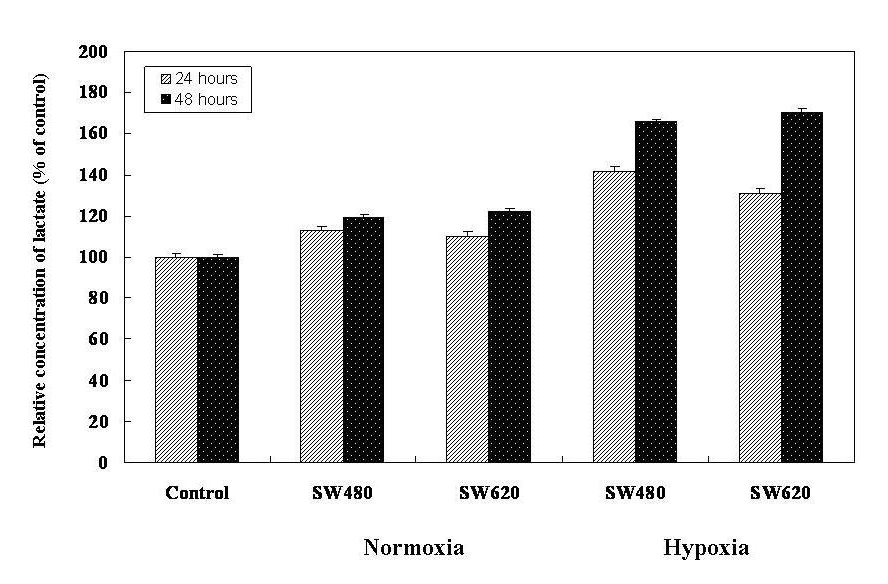
**Relative lactate concentration curves of two colorectal cancer cell lines**. Supernatants from the two colorectal cancer cell lines SW480 and SW620. Control specimens were acquired from the supernatants of culture medium only. The concentrations of lactate were examined after incubation for 24 h and 48 h in hypoxia and normoxia conditions. Relative elevated lactate concentrations of SW480 cell lines were 3.24-fold (hypoxia/normoxia: 41.63/12.84; after 24 h incubation) and 3.36-fold (hypoxia/normoxia: 65.70/19.56; after 48 h incubation); however, there were 3.06-fold (hypoxia/normoxia: 30.78/10.05; after 24 h incubation) and 3.17-fold (hypoxia/normoxia: 70.45/22.23; after 48 h incubation) elevated lactate in SW620 cell lines. Values are means ± SD of 3 independent experiments with triplicate dishes.

The concentration of lactate is stimulated differently by hypoxia and normoxia after 24 and 48 h, respectively. Relative lactate concentration curves of the two colorectal cancer cell line supernatants, SW480 and SW620, are shown in Figure 2. The relative elevated lactate concentrations of SW480 cell lines were 3.24-fold (hypoxia/normoxia: 41.63/12.84; after 24 h incubation) and 3.36-fold (hypoxia/normoxia: 65.70/19.56; after 48 h incubation); however, lactate was elevated 3.06-fold (hypoxia/normoxia: 30.78/10.05; after 24 h incubation) and 3.17-fold (hypoxia/normoxia: 70.45/22.23; after 48 h incubation) in SW620 cell lines.

### HIF-1α and GLUT1 mRNA expressions in SW480 and SW620 cell lines under normoxia and hypoxia conditions

**Figure 3:**
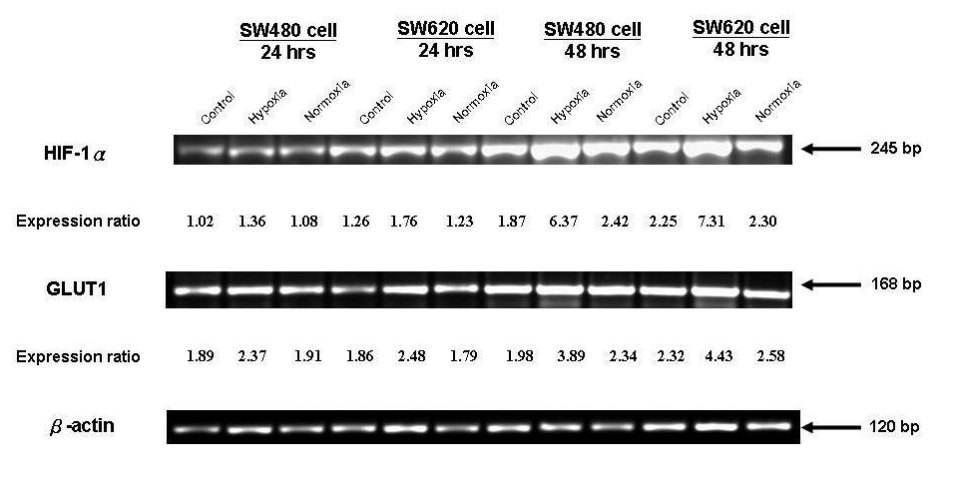
**RT-PCR analysis of HIF-1α and GLUT1 mRNA expression in SW480 and SW620 cell lines under normoxia and hypoxia conditions**. Results of RT-PCR analysis of HIF-1α mRNA and GLUT1 mRNA expression in SW480 and SW620 cell lines incubated for approximately 24 and 48 h in hypoxia and normoxia conditions. The relative expression ratio of HIF-1α mRNA and GLUT1 mRNA under hypoxia conditions were all higher than normoxia in both cell lines. The mRNA levels of the housekeeping gene β-actin served as the internal control for the normalization of mRNA levels in experimental samples. The measured values in a series were normalized to the β-actin signal of the respective control.

For the characterization of the molecular effects in response to hypoxic conditions in all investigated cells, quantitative RT-PCR analysis of HIF-1α and GLUT1 was performed. The mRNA levels of the housekeeping gene β-actin served as the internal control for the normalization of mRNA levels in experimental samples. Results of RT-PCR analysis of HIF-1α mRNA and GLUT1 mRNA expression in SW480 and SW620 cell lines which were incubated for approximately 24 and 48 h respectively in hypoxia conditions and normoxia conditions are shown in Figure 3. The relative expression ratios of HIF-1α mRNA and GLUT1 mRNA under hypoxia were all higher than normoxia conditions in both two cell lines.

**Figure 4:**
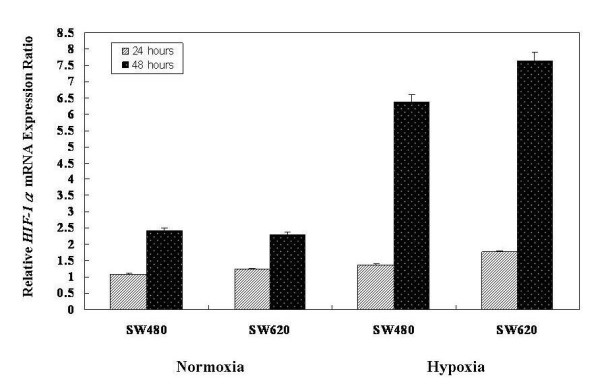
**Comparison of the relative HIF-1α mRNA expression ratio of SW480 and SW620 cell lines between normoxia and hypoxia conditions**. The results showed that the relative HIF-1α mRNA expression levels were elevated in cells exposed to hypoxia conditions. The relative HIF-1α mRNA expression ratio of SW480 cell lines were 1.26-fold (hypoxia/normoxia: 1.36/1.08; after 24 h incubation) and 2.63-fold (hypoxia/normoxia: 6.37/2.42; after 48 h incubation); however, there was a 1.43-fold (hypoxia/normoxia: 1.76/1.23; after 24 h incubation) and 3.32-fold elevated HIF-1α mRNA expression (hypoxia/normoxia: 7.63/2.30; after 48 h incubation) in SW620 cell lines. The control groups were incubated in humidified atmospheric air without the addition of CO_2_. Values shown are means ± SD of 3 independent experiments with triplicate specimens.

Results of the comparison of the relative HIF-1α mRNA expression ratio of SW480 and SW620 cell lines between normoxia and hypoxia conditions are shown in Figure 4. The relative HIF-1α mRNA expression levels were elevated while cells were exposed to hypoxia conditions. The relative HIF-1α mRNA expression ratios of SW480 cell lines were 1.26-fold (hypoxia/normoxia: 1.36/1.08; after 24 h incubation) and 2.63-fold (hypoxia/normoxia: 6.37/2.42; after 48 h incubation); however, 1.43-fold (hypoxia/normoxia: 1.76/1.23; after 24 h incubation) and 3.32-fold elevated HIF-1α mRNA expressions (hypoxia/normoxia: 7.63/2.30; after 48 h incubation) were observed in SW620 cell lines.

**Figure 5:**
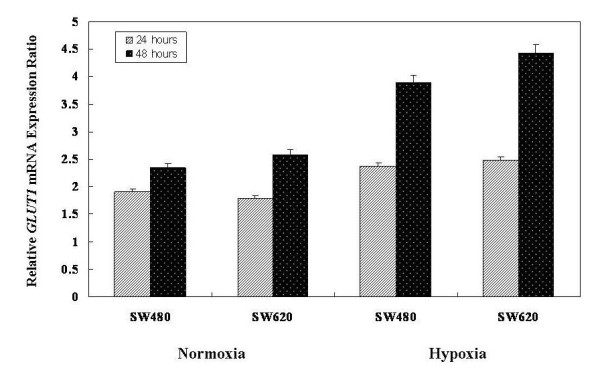
**Comparison of the relative GLUT1 mRNA expression levels of SW480 and SW620 cell lines between normoxia and hypoxia conditions**. The results showed that the relative GLUT1 mRNA expression level was elevated in cells exposed to hypoxia. The relative GLUT1 mRNA expression ratio of SW480 cell lines were 1.24-fold (hypoxia/normoxia: 2.37/1.91; after 24 h incubation) and 1.66-fold (hypoxia/normoxia: 3.89/2.34; after 48 h incubation); however, there was a 1.39-fold (hypoxia/normoxia: 2.48/1.79; after 24 h incubation) and a 1.72-fold elevated GLUT1 mRNA expression (hypoxia/normoxia: 4.43/2.58; after 48 h incubation) in SW620 cell lines. The control groups were incubated in humidified atmospheric air without the addition of CO_2_. Values shown are means ± SD of 3 independent experiments with triplicate specimens.

A comparison of the relative GLUT1 mRNA expression levels of SW480 and SW620 cell lines between normoxia and hypoxia conditions is illustrated in Figure 5. The results showed that the relative GLUT1 mRNA expression levels were elevated when cells were exposed to hypoxia. The relative GLUT1 mRNA expression ratio of SW480 cell lines were 1.24-fold (hypoxia/normoxia: 2.37/1.91; after 24 h incubation) and 1.66-fold (hypoxia/normoxia: 3.89/2.34; after 48 h incubation). In SW620 cell lines, however, we observed 1.39-fold (hypoxia/normoxia: 2.48/1.79; after 24 h incubation) and 1.72-fold elevation of GLUT1 mRNA expression (hypoxia/normoxia: 4.43/2.58; after 48 h incubation).

### Glycolysis- and hypoxia-associated gene expressions in CRC tissue specimens

We used mRNA membrane array to explore gene expression profiles in CRC tissues. Seven candidate genes of glycolysis-associated genes (i.e., HK1, GPI, PGK1, PGK2, ENO2, PKM2, and GLUT1) and nine hypoxia-associated genes (i.e., uPA, CA9, CA12, TP, bFGF, COX-2, HIF-1α, HIF-2α, and VEGF) were detected in the present study. We spotted the well-designed oligonucleotide for each candidate gene onto nylon membrane for further analysis (Figure 1). Membrane array analysis was performed on 10 human CRC tissue samples and 10 paired human normal colorectal tissue samples. Additional File 4 lists the relative gene expression ratios of colorectal cancer tissues compared with normal controls.

We found that GLUT1, HIF-1α, and HIF-2α genes presented 100% overexpression in these 10 CRC tissue samples. The overexpression rate of the other genes were 60% in HK1; 70% in GPI and PGK1; 80% in ENO2, PKM2, uPA, TP, bFGF and VEGF; and 90% in PGK2, CA9, CA12 and COX-2. The overexpression ratios of GLUT1, HIF-1α and HIF-2α genes in poorly differentiated stage-III and -IV samples were higher than in well differentiated stage-I and moderately differentiated stage-II samples (Additional File 4).

**Figure 6:**
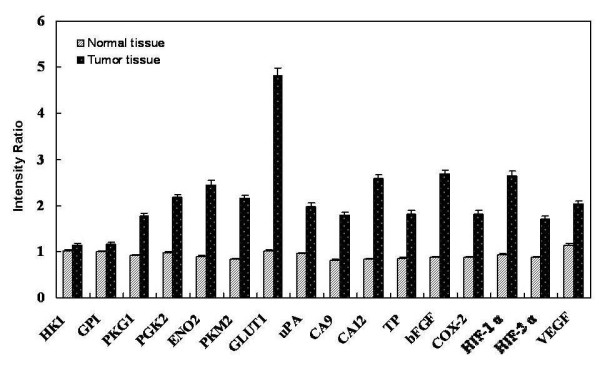
**Comparison of the hypoxia- and glycolysis-associated gene expression intensity between human CRC (Case No. 6) and normal tissue**. The results showed the relative expression ratio of sixteen genes. The overexpression of GLUT1, HIF-1α, and HIF-2α are presented in these human colorectal cancer tissue samples.

In Figure 1B, we compared the gene expression intensity ratio obtained by membrane array between human CRC tissue specimens (Case No. CRC6) and normal control. The results show the expression ratios for the sixteen genes, including 1.11 in HK1, 1.17 in GPI, 1.94 in PGK1, 2.24 in PGK2, 2.75 in ENO2, 2.59 in PKM2, 4.73 in GLUT1, 2.08 in uPA, 2.17 in CA9, 3.07 in CA12, 2.12 in TP, 3.04 in bFGF, 2.09 in COX-2, 3.55 in HIF-1α, 3.73 in HIF-2α, and 1.78 in VEGF. In the poorly differentiated stage-IV colon adenocarcinoma specimen (Case No. CRC6), we found that GLUT1 had the highest overexpression of the glycolysis-pathway-associated genes (Figure 6).

### Glycolysis- and hypoxia-associated gene expressions in the peripheral blood specimens of 100 CRC patients

We used mRNA membrane array to explore gene expression profiles in CRC peripheral blood specimens. The highest overexpressed gene of the glycolysis-pathway-associated genes, GLUT1, and five hypoxia-associated genes (i.e., CA9, CA12, COX-2, HIF-1α, and HIF-2α) were detected in the present study. The overexpression rates of all five genes were higher than 90% in human CRC tissue samples. The correlation between mRNA expressions and clinicopathological features of CRC patients are shown in Additional File 5.

No statistical correlations between CA9, CA12, COX-2, HIF-1α, and HIF-2α overexpression and age or sex (*P *> 0.05; Additional File 5) were found. However, advanced cancer stage was significantly related to GLUT1 overexpression (*P *= 0.004). The GLUT1 overexpression rates for UICC stage I, II and III were 33.3%, 85.9% and 86.5%, respectively. The risk of GLUT1 overexpression was higher in stage II and III than in stage I (RR: 12.482; *P *= 0.001).

## Discussion

Colorectal cancer (CRC) is a leading cause of morbidity and mortality in Europe and the United States [26-28]. As several investigators have reported, approximately 40% to 50% of CRC patients who undergo a supposedly curative resection, subsequently develop metastatic disease and die of their disease within 5 years [29,30]. Despite the efforts to clarify the correlation between CRC prognosis and gene expression by previous investigators, the results have been equivocal [31,32].

Hypoxia has a strong influence on the metabolism of cells and, as a consequence, on the physiology and vasculature of solid tumors. One key regulator of hypoxia – the transcription factor HIF-1 – has been shown to be involved in the up-regulation of many genes which are involved in erythropoiesis, angiogenesis, glycolysis, glucose transport, and several other functions [33,34]. Apart from activating HIF-1, hypoxia stimulates a great variety of cellular responses. Many signaling pathways that are deregulated in cancer promote HIF-1 activity.

The main objective of this study was to discover a molecular marker that could be used to assess the hypoxia level of peripheral circulating cancer cells in the blood specimens of cancer patients by a well-established membrane array method [22,25,35]. The mRNA markers employed included seven glycolysis-associated genes (i.e., HK1, GPI, PGK1, PGK2, ENO2, PKM2, and GLUT1) and nine hypoxia-associated genes (i.e., uPA, CA9, CA12, TP, bFGF, COX-2, HIF-1α, HIF-2α, and VEGF). The data obtained revealed that HIF-1α and HIF-2α are the predominant overexpressed hypoxia-associated genes found in CRC tissues. There was 100% mRNA overexpression of GLUT1 gene in our human CRC specimens that paralleled the hypoxic mRNA overexpression (HIF-1α). GLUT1 gene was the significant up-regulated glycolysis-associated gene in hypoxic CRC cells.

Malignant cells are known to have accelerated metabolism, high glucose requirements, and increased glucose uptake. Although several studies have demonstrated that cancer cells have increased glycolytic activity in many malignant diseases *in vivo *and *in vitro *[36-39], participation of the glycolytic pathway and glycolysis-associated genes in tumorigenesis of human CRC has yet to be elucidated. In our study, we concluded that hypoxia can stimulate glycolysis of CRC cells. The relative lactate concentration under hypoxic conditions is 3.24- to 3.36-fold elevated in SW480 cells, and 3.06- to 3.17-fold in SW620 cells when compared with normoxic conditions, observed up to 48 h.

Of late, interest in glucose uptake by tumor cells has increased. Facilitative glucose transporters (GLUTs) allow the energy-independent transport of glucose across the hydrophobic cell membrane, downwards along its concentration gradient. Each of the glucose transporter proteins possesses different affinities for glucose and other hexoses, such as fructose. GLUT1, GLUT3, and GLUT4 have a high affinity for glucose, allowing transport of glucose at a high rate under normal physiological conditions [40,41]. GLUT1 is present at variable levels in many tissues and is believed to be responsible for basal glucose uptake [42,43]. We performed RT-PCR analysis of HIF-1α and GLUT1 mRNA expressions in SW480 and SW620 CRC cell lines. The relative expression ratio of HIF-1α mRNA and GLUT1 mRNA in hypoxic conditions was higher than in normoxic conditions. In the hypoxic condition, HIF-1α mRNA expression ratio were 1.26- to 2.63-fold elevated in SW480 cells and 1.43- to 3.32-fold higher in SW620 cells. GLUT1 mRNA expression ratios were similarly proportional to the hypoxic conditions with 1.24- to 1.66-fold elevation in SW480 cells and 1.39- to 1.72-fold elevation in SW620 cells. SW480 and SW620 represented the two human colorectal adenocarcinoma cell lines of UICC II and UICC III, respectively. We found that GLUT1 mRNA expression could be stimulated by hypoxia, and the expression is more predominant in the advanced CRC cell lines (UICC III) *in vitro*. Malignant cells exhibit increased glycolytic metabolism. Warburg first established a correlation between aggressive tumor phenotypes and elevated glycolysis when he observed that many tumors produce excessive lactate in the presence of oxygen [44]. Previous reports observed that suppression of facilitative GLUT1 mRNA could suppress the growth of gastric cancer cell line (MKN45) [45]. Thus, an increased uptake of glucose provides energy to enhance cell synthesis. This might be an important aspect of malignant transformation and uncontrolled cell growth.

GLUT1 expression was also elevated in other tumor entities. In 2007, Shao *et al*. suggested that the abnormal expression of GLUT1 may perhaps participate in serous ovarian tumor occurrence and development and may be considered as a marker reflecting tumor malignant behaviour [46]. Bone and soft tissue sarcomas with GLUT1 overexpression showed significantly poor OS compared with those without GLUT1 overexpression (*P *= 0.029) according to Endo *et al*. [47]. In 2008, Ciampi *et al*. reported that GLUT1 and GLUT3 were the most important glucose transporters in the thyroid tumoral cells. In particular, GLUT1 was the most prevalent in less-differentiated cells, while GLUT3 was the most prevalent in well-differentiated cells [48]. HIF-1α was increasingly expressed from early stages through advanced stages of endometrioid adenocarcinoma, paralleled by activation of its downstream genes, such as GLUT1, vascular endothelial growth factor (VEGF) and increased angiogenesis. These results highlight the importance of hypoxia and related pathways in the progression of endometrial carcinoma [49]; however, there was a contradictory report on the expression of GLUT1 under hypoxic conditions. In a series of studies, Mayer *et al*. [50] examined the expression of HIF-1α and GLUT1 in tissue micro-areas where direct O_2 _measurements had previously been carried out, so that the influence of tumor heterogeneity could be reduced to a minimum. Using this methodology, no correlation between the expression of HIF-1α and GLUT1 and direct pO_2 _measurements could be found.

Little is known about the details of CRC tumorigenesis. Knowing how to rapidly organize new techniques for exploring the prognosis is a key to providing physicians with the information needed for further intervention. In the present study, we found GLUT1 mRNA expression in the peripheral blood of CRC patients. Advanced cancer stage was significantly related to GLUT1 overexpression (*P *= 0.004), the risk of which was higher in stages II and III than in stage I (RR: 12.482; *P *= 0.001). GLUT1 overexpression in the blood also correlated with advanced tumor stages. The GLUT1 overexpression rates for stages I, II and III were 33.3%, 85.9% and 86.5%, respectively. This finding suggests that GLUT1 may be a stage-related marker that could be determined by a non-invasive method. However, the low number of patients in stage I would be over-interpreted according to the significant *P *value of GLUT1 in Additional File 5. The additional glycolysis- and hypoxia-associated gene expression data obtained from peripheral blood showed that no statistical correlations existed between CA9, CA12, COX-2, HIF-1α, and HIF-2α overexpression and age or sex (*P *> 0.05). In 2008, Nguyen *et al*. found that high correlations between the primary tumours and metastatic lymph nodes in non-small-cell lung cancer patients and the GLUT1 mediated fluorodeoxyglucose (FDG) uptake may be useful for mediastinal lymph nodes discrimination by fluorodeoxyglucose positron emission tomography (FDG-PET). It might be possible to combine GLUT1 with other new image diagnostic techniques (e.g., FDG-PET) to interpret the clinical condition of tumours [51].

## Conclusion

In conclusion, the levels of HIF-1α, HIF-2α, and the GLUT1 genes were significantly overexpressed in CRC tissue specimens, and the elevated ratio of GLUT1 was greater in stage-III and -IV CRC tissue specimens than in stage-I and -II specimens. The GLUT1 mRNA was also more increased in the peripheral blood of stages-II and -III CRC patients than in stage-I patients. The overexpression ratio of GLUT1 gene is higher in poorly differentiated stage-III and -IV human CRC specimens. We postulate that the overexpression of GLUT1 may play an important role in the tumour progression of CRC. GLUT1 should be considered to have potential carcinogenesis potency in advanced human CRC. In further studies, GLUT1 expression should be investigated as a useful target for therapeutic intervention. Future research will use this molecular marker to identify those cancer patients who would respond best to radiation therapy and chemotherapy, thereby improving the clinical treatment outcome.

## Competing interests

The authors declare that they have no competing interests.

## Authors' contributions

FYC and MYH contributed equally to the manuscript and both are the first author. FYC and MYH analyzed the data and drafted the manuscript. FYC, MYH, CSY, JYW, TLC, LCY and SCL made contributions in data acquisition, molecular genetic analyses, statistical analyses and data interpretation. FYC, MYH and SRL participated in the design and coordination of the study.

## Pre-publication history

The pre-publication history for this paper can be accessed here:

http://www.biomedcentral.com/1471-2407/9/241/prepub

## Supplementary Material

Additional file 1 **Table 1**. Sequences for primers for RT-PCR Click here for file

Additional file 2 **Table 2**. Click here for file

Additional file 3 Table 3 Click here for file

Additional file 4 Table 4 Click here for file

Additional file 5 Table 5 Click here for file
