# Supplementary figures and images for: GLUT1 gene is a potential hypoxic marker in colorectal cancer patients
Source: BMC Cancer. 2009 Jul 20;9:241. doi: 10.1186/1471-2407-9-241 (PMC3087329; doi:10.1186/1471-2407-9-241)

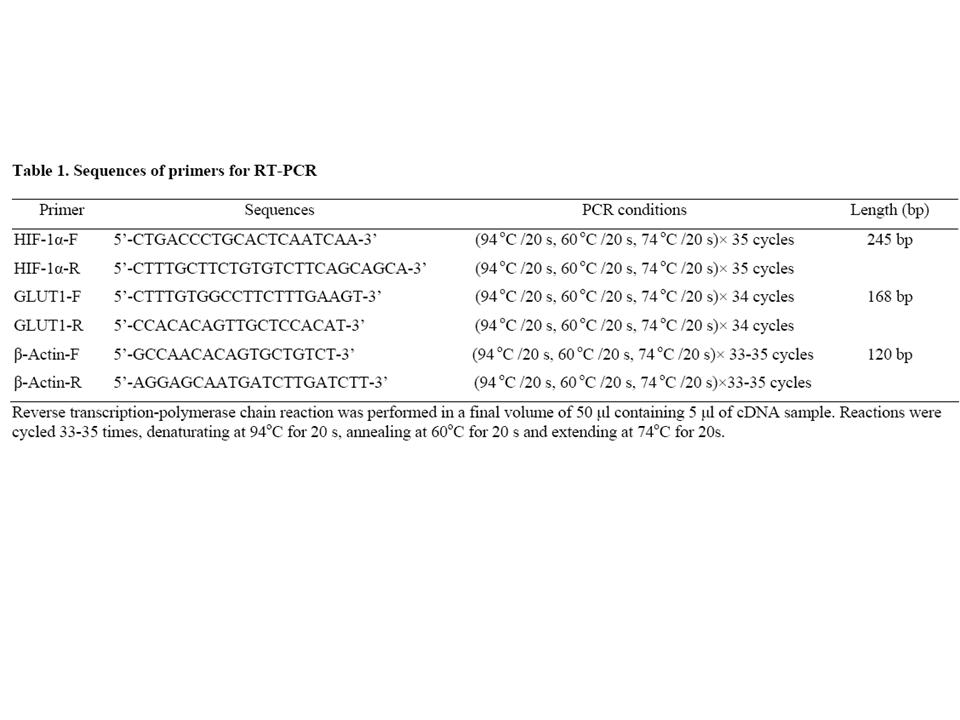

Supplement: Additional file 1 — Table 1. Sequences for primers for RT-PCR [file 1471-2407-9-241-S1.jpeg]

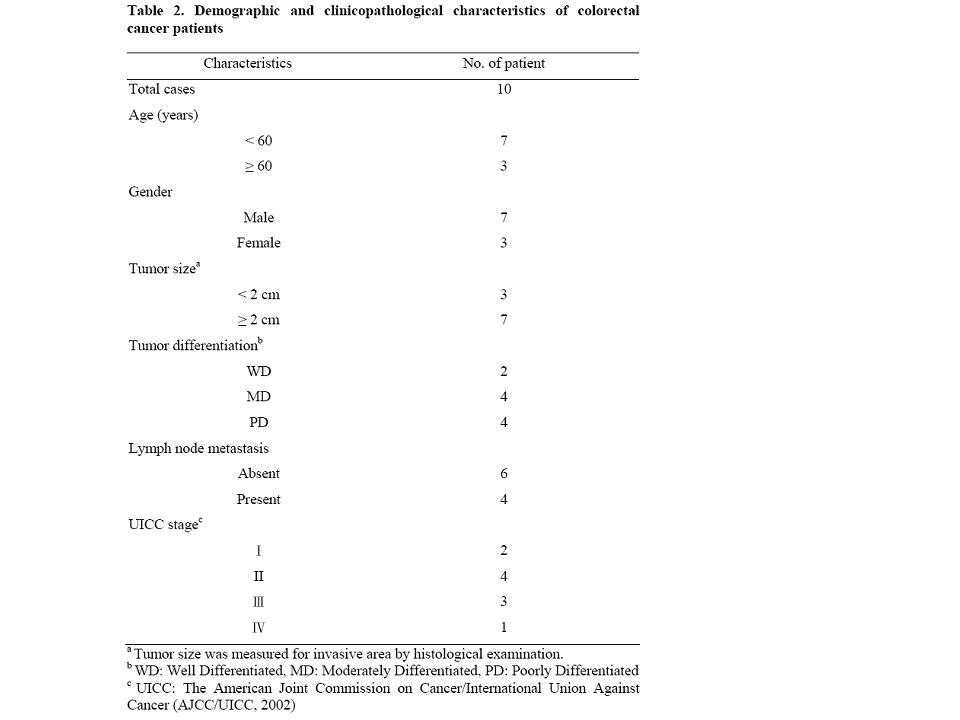

Supplement: Additional file 2 — Table 2. [file 1471-2407-9-241-S2.jpeg]

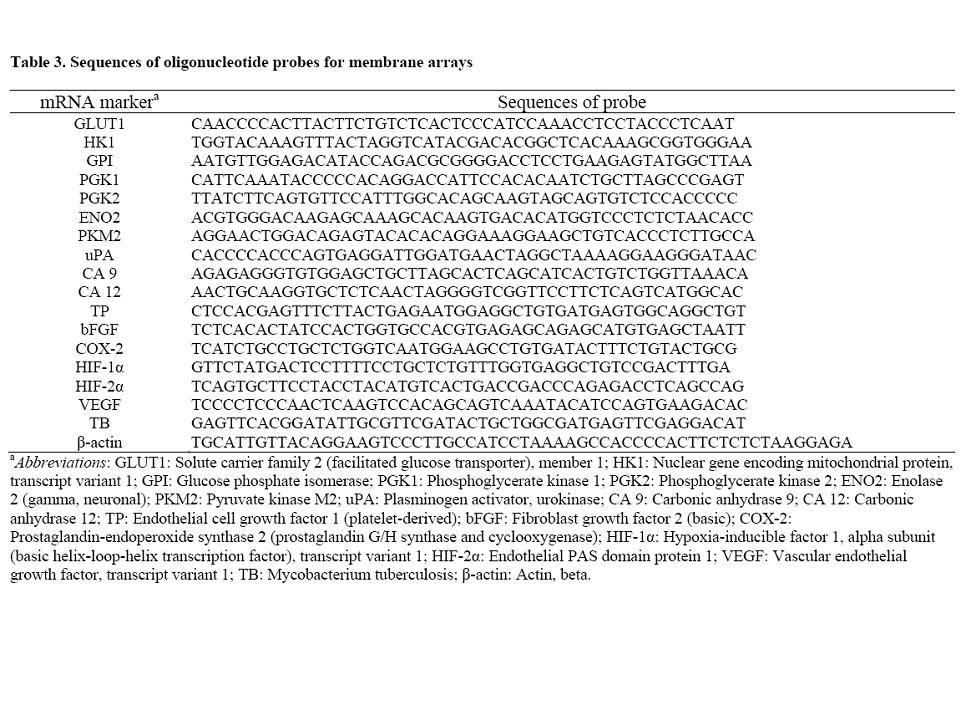

Supplement: Additional file 3 — Table 3 [file 1471-2407-9-241-S3.jpeg]

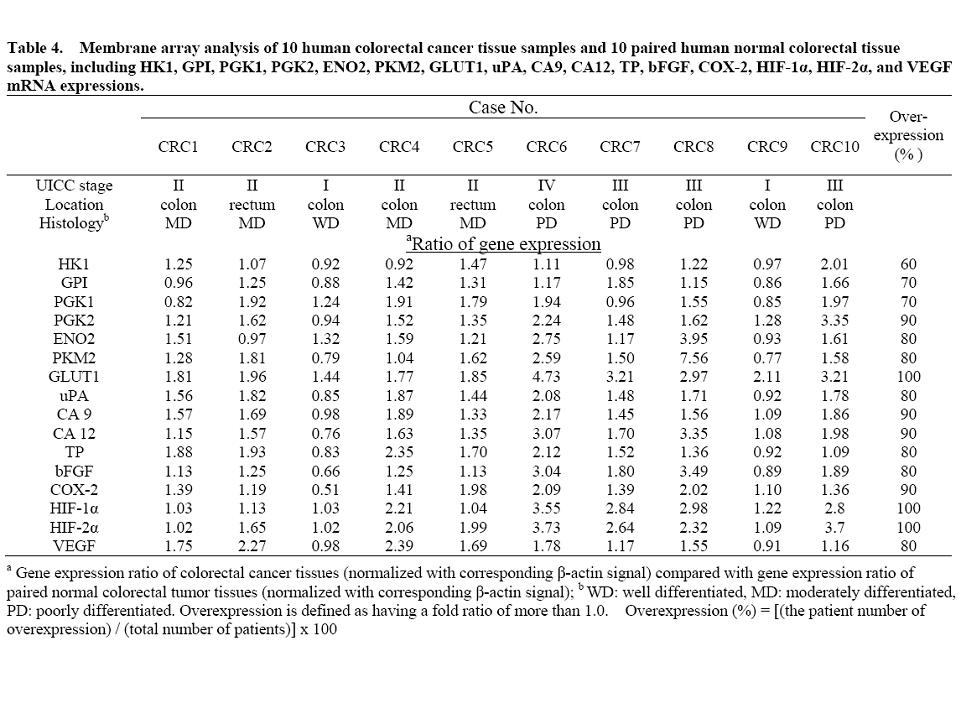

Supplement: Additional file 4 — Table 4 [file 1471-2407-9-241-S4.jpeg]

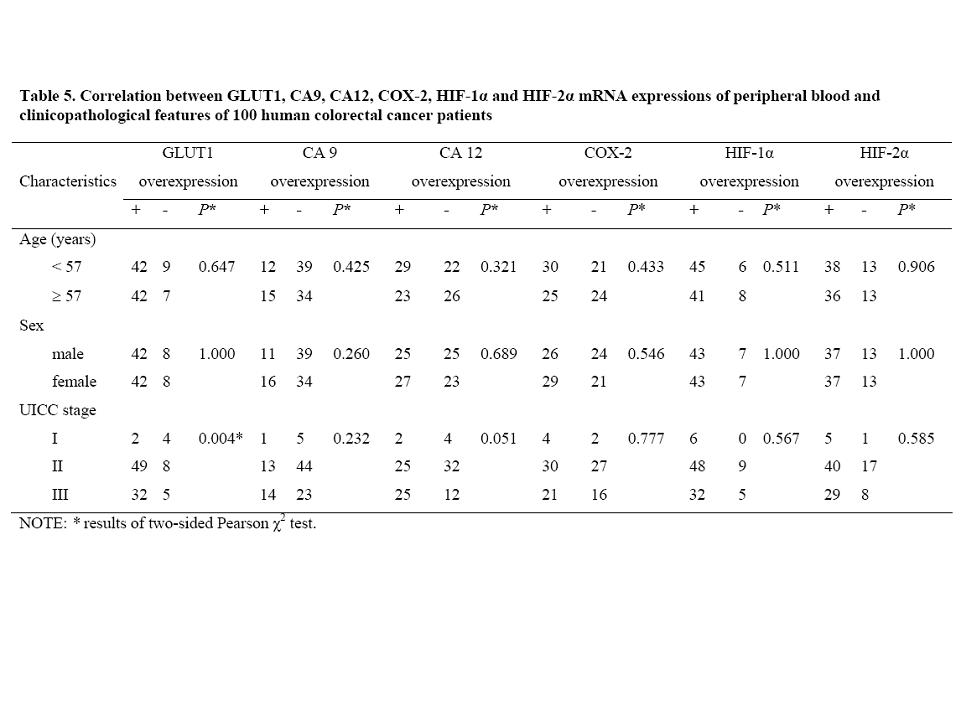

Supplement: Additional file 5 — Table 5 [file 1471-2407-9-241-S5.jpeg]
